# Real‐time longitudinal analysis of human gliomas reveals in vivo genome evolution and therapeutic impact under standardized treatment

**DOI:** 10.1002/ctm2.956

**Published:** 2022-07-08

**Authors:** Sensen Xu, Zhiyuan Sheng, Jinliang Yu, Kaiyuan Deng, Shuang Wu, Yage Bu, Guangzhong Guo, Ziyue Zhang, Guanzheng Liu, Yushuai Gao, Zhaoyue Yan, Chaojie Bu, Yingkun He, Gang Liu, Ajmal Zemmar, Juha Hernesniemi, Lingfei Kong, Meiyun Wang, Tianxiao Li, Xingyao Bu

**Affiliations:** ^1^ Department of Neurosurgery, Juha International Center for Neurosurgery Zhengzhou University People's Hospital, Henan Provincial People's Hospital, Henan University People's Hospital Zhengzhou Henan China; ^2^ Department of Psychological Medicine Zhengzhou University People's Hospital, Henan Provincial People's Hospital, Henan University People's Hospital Zhengzhou Henan China; ^3^ Henan Provincial Neurointerventional Engineering Research Center Henan International Joint Laboratory of Cerebrovascular Disease, Henan Engineering Research Center of Cerebrovascular Intervention Innovation Zhengzhou Henan China; ^4^ Department of Cerebrovascular Disease Zhengzhou University People's Hospital, Henan Provincial People's Hospital, Henan University People's Hospital Zhengzhou Henan China; ^5^ Department of Center for Clinical Single Cell Biomedicine, Clinical Research Center, Department of Oncology Zhengzhou University People's Hospital, Henan Provincial People's Hospital, Henan University People's Hospital Zhengzhou Henan China; ^6^ Department of Pathology Zhengzhou University People's Hospital, Henan Provincial People's Hospital, Henan University People's Hospital Zhengzhou Henan China; ^7^ Department of Radiology Zhengzhou University People's Hospital, Henan Provincial People's Hospital, Henan University People's Hospital Zhengzhou Henan China

Dear Editor,

1

The molecular characterization of glioma genes is essential for properly managing and treating this heterogeneous disease.^1^, ^2^ However, the true in vivo evolution of the genome of glioma patients during treatment and its impact on clinical outcomes is unclear.^3^ Here, we have performed an in‐depth analysis of ctDNA from patients’ tumour in situ fluid (TISF)^4^ samples at baseline (BL), disease progression, and post‐disease progression and elucidate the value of molecular markers such as GNAS (guanine nucleotide binding protein, alpha stimulating) complex locus/CIC (capicua transcriptional repressor) and maximum variant allele frequencies (MVAF) in assessing tumour progression and patient prognosis under therapeutic pressure.

The study flow chart for this study is shown in Figure S1. In total, 33 patients with glioma were recruited; all safely had maximum tumour resection and received standardized chemotherapy with temozolomide and a combination of temozolomide and bevacizumab after imaging suggested disease progression. Statistics on these patients with primary tumours and TISF at disease progression under standardized treatment are presented in Table S1. In the primary tumour of 33 patients, gene amplification occurred in 33.3% (11/33) patients (Figure S2A), and high‐frequency‐mutated genes were TP53, PTEN, IDH1, NF1, and so on (Figure S2B).

We compared TISF at disease progression with paired primary tumour samples in 33 patients (Figure 1A), and further analysis showed that, to some extent, TISF at disease progression was weakly correlated with the corresponding somatic variants in the primary tumour tissue (Figure 1B). However, in two patients with recurrent reoperation, there was strong concordance and correlation between the recurrent tissue and the corresponding mutated gene in the TISF at disease progression (Figures 1D,E and S3A,B). Moreover, there is a clear difference in the mutational status of top mutated genes like GNAS/CIC between TISF at disease progression and primary tumour (Figure 1C). These suggest that, to some extent, TISF is reliable for detecting somatic variants during systemic treatment of gliomas and can be used as an alternative to tissue analysis and to obtain accurate genomic information on the recurrent tumour.^5^

**FIGURE 1:**
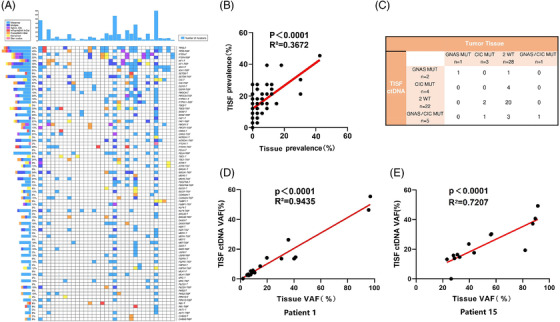
Consistency analysis of mutations in tumour in situ fluid (TISF) and primary tumour tissue samples paired at disease progression in 33 patients. (A) Genomic analysis of some high‐frequency mutations between the primary tumour tissue samples and the TISF samples of disease progression. The top column represents the number of mutations carried by patients; the side columns represent the number of patients carrying a particular mutation. Here, more abundant tumour DNA was detected in the TISF compared to the tumour tissue. (B) Correlation analysis between the frequency of mutations in 68 individual genes in ctDNA samples from TISF at disease progression and primary tumour tissue samples (*R* ^2^ = .3672; *p* < .0001, Spearman rank correlation). (C) Comparison of GNAS and CIC mutation status in primary tumour tissue samples and recurrent TISF samples. The mutation rate in GNAS tumour tissue was 6.1% (*n* = 2) and in TISF was 21.2% (*n* = 7), and CIC mutation rates were 12.1% (*n* = 4) in tumour tissue and 27.3% (*n* = 9) in TISF. Furthermore, the overall concordance of GNAS/CIC status between TISF and the primary tumour tissue at the time of disease progression was 66.7% (22/33). In comparison, the inconsistency rates of GNAS and CIC status were 15.6% (5/33) and 27.3% (9/33), respectively. (D and E) Correlation analysis between DNA samples from a recurrent secondary surgical tumour tissue and the variant allele fraction (VAF) of the corresponding gene in the ctDNA sample in the TISF at the time of at disease progression (Patient 1: *R* ^2^ = .9435, *p* < .0001; Patient 15: *R* ^2^ = .7207, *p* < .0001, Spearman's rank correlation). MUT, mutant; T, tissue; WT, wild type

In the cohort of 20 patients (time from patients’ postoperative period to imaging suggestive of progressive disease (PD), shown in Figure S2C) with serial TISF samples collected prior to PD, we compared the 20 patients with top mutated genes between BL and last TISF before PD, showing that most patients had a significantly altered number of mutated genes at PD (Figure 2A,B,E), with a significant change in clearance and acquisition rates of top mutated genes like GNAS/CIC (Figure 2C), and generating an actionable target map for postoperative gliomas (Figure 2D). Furthermore, we found that MVAF was also differentially increased or decreased in TISF at PD compared to BL (Figure 2E), with more significant changes in VAF < 1% in genes with an increased number of mutations (Figure 2F). These changes can reflect the high temporal heterogeneity of the glioma genomic landscape during treatment, the different forms of recurrence (Figure S4), and the fact that there are many potential precision therapeutic targets for clinical intervention.

**FIGURE 2:**
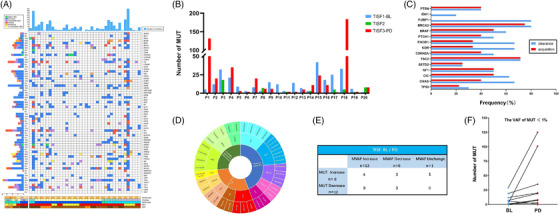
Genomic temporal heterogeneity of ctDNA in tumour in situ fluid (TISF) under standard normative treatment in 20 patients. (A) Genomic analysis of mutations in gliomas between TISF samples at baseline (BL) and disease progression. The top column represents the number of mutations carried by patients; the side columns represent the number of patients carrying a particular mutation; and the bottom column represents patient characteristics, including age, gender, WHO classification, tumour staging, and sample type. (B) Changes in the number of mutated genes in TISF from BL to disease progression. We found that the number of mutations is highly variable during tumour evolution, with 60% (12/20) of patients having a reduced number of mutated genes and 40% (8/20) having an increased number of mutated genes at progressive disease (PD) compared to TISF at BL. (C) Clearance and acquisition rates of high‐frequency mutant genes under standard regulatory treatment pressure. Overall, 66.7% (6/9) of patients with GNAS mutations showed GNAS mutation clearance, and of these, ctDNA was detectable in TISF in all patients showing GNAS clearance. Overall, 62.5% (5/8) of patients with CIC mutations showed CIC mutation clearance, whereas 40% (2/5) and 50% (3/6) of patients showed new GNAS and CIC mutations, respectively. (D) Ongoing international clinical trials for glioma and FDA/NMPA approved actionable targets for other tumours. The outer ring represents targeted agents used for actionable targets. The middle ring represents mutated genes that can be used as targets. The inner ring represents currently ongoing glioma‐related clinical trials and targeted agents FDA/NMPA approved for use in other tumours. (E) Comparison of different states of maximum variant allele frequencies (MVAF) and number of mutated genes in TISF at BL and PD. Overall, 20% (4/20) showed an increase in both MVAF and the number of mutated genes; 45% (9/20) of patients showed an increase in MVAF and a decrease in the number of mutations, with five patients having the same mutated genes as the highest variant allele fraction (VAF) in BL and PD, namely, GNAS, EGFR, H3F3A, PTEN, and PDGFRA; another three patients showed a decrease in both MVAF and number of mutations, and one patient showed no change in MVAF and an increase in the number of mutations. (F) Change in the number of VAF < 1% mutated genes in TISF at BL and PD. These VAF smaller mutated genes exhibit a subclonal structure within the tumour during progression and suggest hypermutation during treatment pressure‐induced tumour progression, mostly involving temozolomide treatment‐related induced mutations (C > T/G > A transition mutations during DNA replication), as a manifestation of treatment response. MUT, mutant

We grouped the 20 patients described earlier according to their different mutational statuses in GNAS/CIC at PD. We found that the clinical prognosis of the patients differed significantly between the different groups. Median progression‐free survival was significantly lower in GNAS/CIC‐cleared patients than patients who acquired or maintained the GNAS/CIC mutation at PD. Furthermore, under treatment pressure, patients with GNAS wild type from BL to PD had a better prognosis compared to patients with GNAS clearance. At the same time, there was no significant difference in prognosis compared to patients who acquired or still maintained the GNAS mutation (Figure 3A,B). Notably, ctDNA levels were positively correlated with tumour load during standard therapy, showing a higher MVAF in TISF when the patients’ tumour volume load was elevated on magnetic resonance imaging (Figure 3C). Furthermore, the median ctDNA level at BL was lower than at PD, indicating that ctDNA levels increased when patients relapsed while on treatment (Figure 3D). These suggest that monitoring ctDNA levels in TISF may be more sensitive in tracking changes in tumour load and predicting tumour recurrence at early ctDNA levels. Primary somatic mutational status of primary tumour tissue may not always be reliable in treatment decisions and prognostic stratification; somewhat, real‐time and subsequent mutational status may significantly impact patient survival and treatment outcome.

**FIGURE 3:**
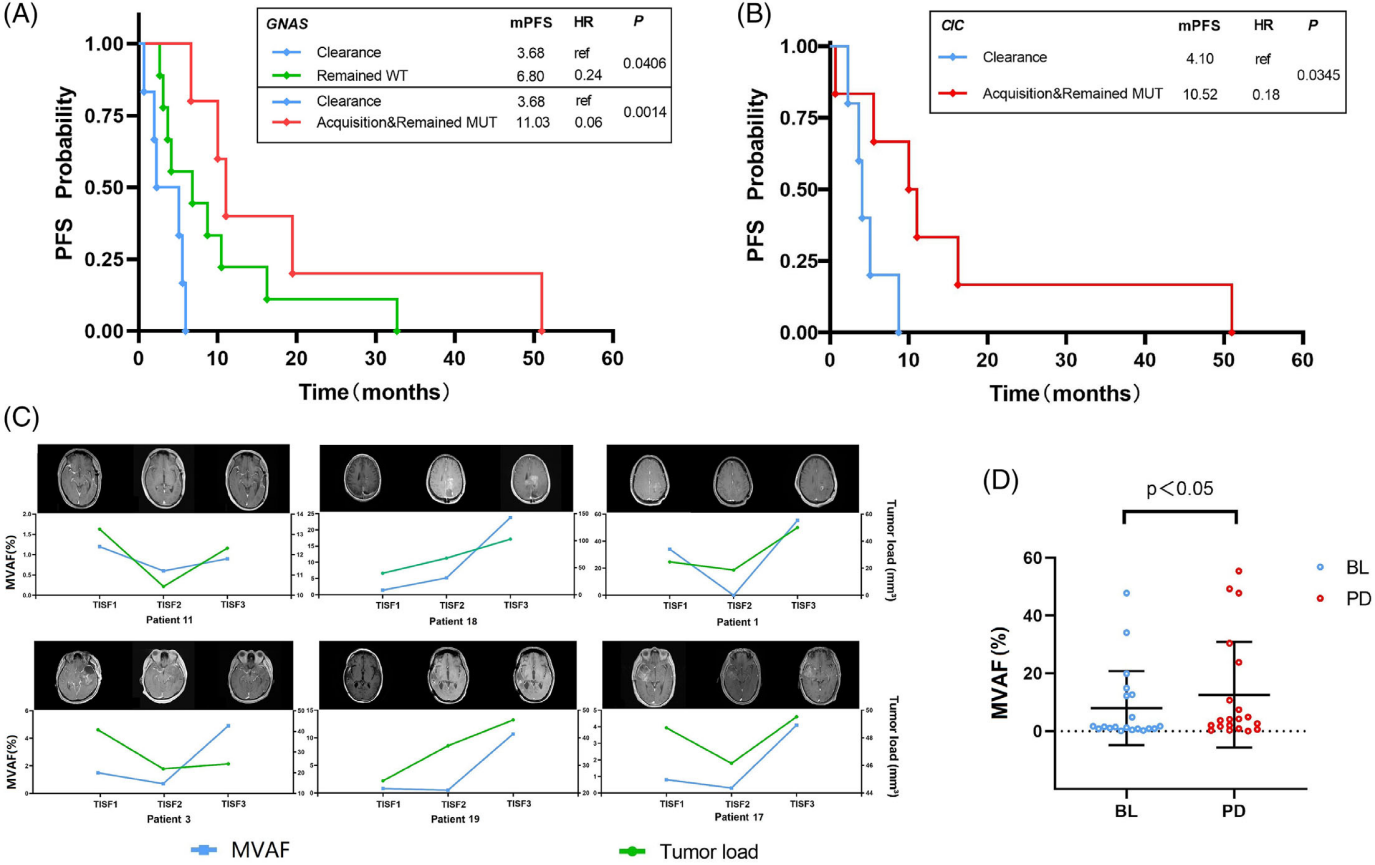
Kaplan–Meier estimates of progression‐free survival (PFS) and the dynamics of ctDNA levels and the relationship with tumour load. (A) and (B) Statistically significant differences in PFS for patients stratified according to different changes in GNAS/CIC status in tumour in situ fluid (TISF) under standard normative therapy (Kaplan–Meier survival curve estimates). Median progression‐free survival (mPFS) was 3.68 and 4.10 months for GNAS and CIC‐cleared patients, respectively, significantly lower than mPFS for patients who acquired or remained with GNAS mutations (11.03 months; *p* = .0014) and CIC mutations (10.52 months; *p* = .0345; Figure 4A) at the time of disease progression. In addition, we observed that under treatment pressure, patients who had been wild type with GNAS from baseline (BL) to progressive disease (PD) had a relatively better prognosis in terms of mPFS of 6.80 months compared to patients with GNAS clearance (*p* = .0406); whereas there was no significant difference in prognosis compared to patients who had acquired or still maintained a GNAS mutation at BL (*p* = .1779). (C) Maximum variant allele frequencies (MVAF) in ctDNA positively correlated with changes in the enhanced fraction of tumour on imaging and tumour load in six patients. (D) Overall, changes in ctDNA levels in TISF at BL and PD. The middle black line is at the mean; the lower and upper half‐lines indicate error lines. The median ctDNA level at BL was 1.45%, which was lower than 3.90% at PD in 20 patients (*p* < .05 ), indicating that ctDNA levels increased when tumour relapsed. MUT, mutant; ref, reference; WT, wild‐type

After disease progression, further analysis of the TISF genome may reveal mechanisms related to bevacizumab's therapeutic effects and drug resistance (Figure 4A). New genetic alterations appeared in 70% (7/10) of patients treated with bevacizumab after treatment, but there was no significant difference in ctDNA levels before and after treatment with bevacizumab (Figure 4D). These changes mainly include reducing the PI3K‐Akt and Ras pathways and the emergence of the cell cycle and HTLV‐I infection pathways (Figure 4B,C). Arya et al. have shown that by analysing the gene expression data of xenografts of tumours of glioblastoma patients, the resistance mechanism involved PI3K‐Akt, cell cycle, and other related signalling pathways.^6^ We obtained similar results using real in vivo genomic data in TISF, suggesting this could be a potential mechanism of treatment and resistance associated with bevacizumab treatment.

**FIGURE 4:**
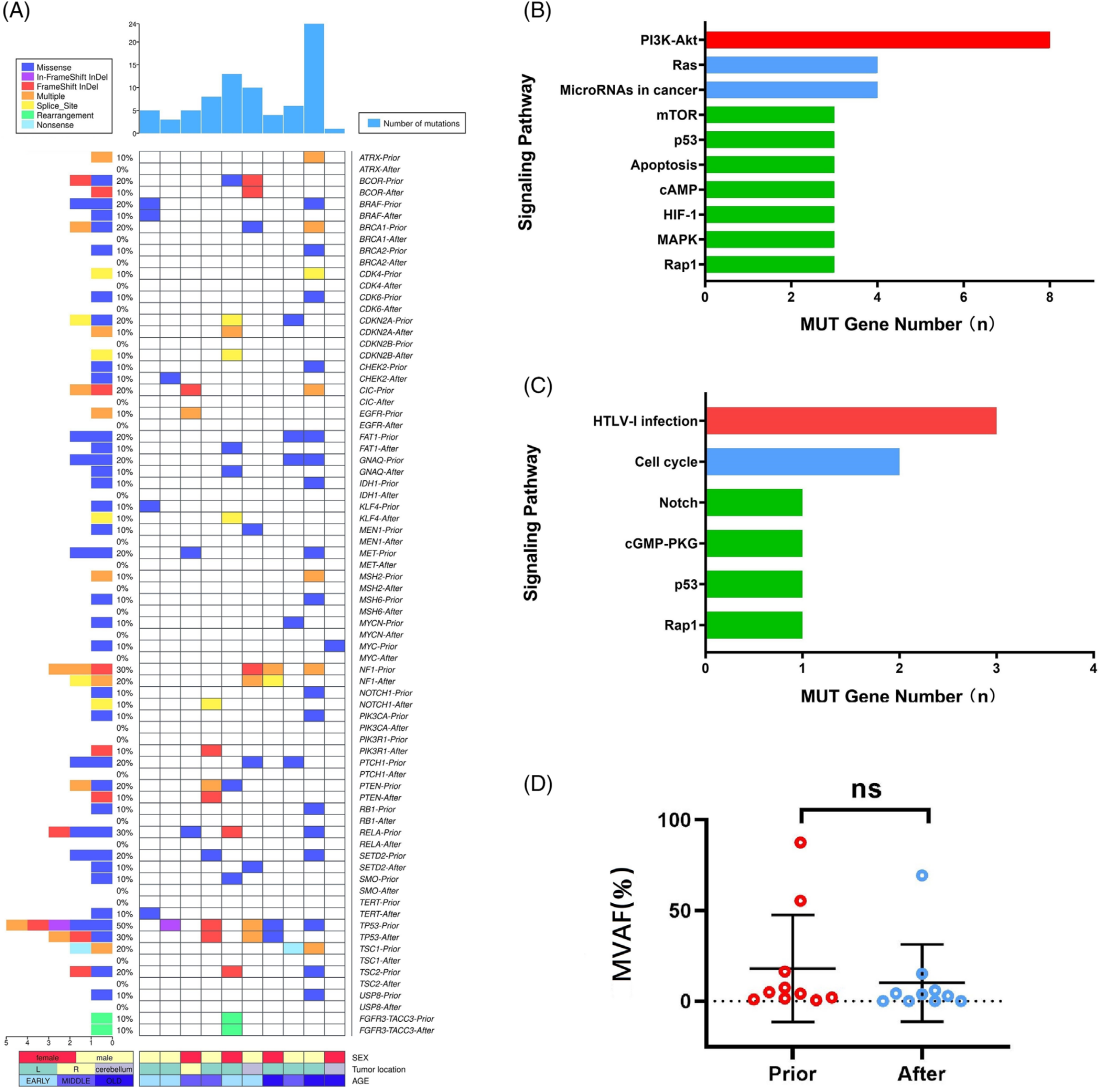
Genomic temporal heterogeneity during chemotherapy with temozolomide combined with bevacizumab after disease progression in 10 patients. (A) Genomic analysis of mutations between tumour in situ fluid (TISF) at progressive disease (PD) and TISF 3 months after adding bevacizumab showed new mutations in 70% (7/10) of patients and negative TISF results in 30% (3/10) of patients; all three of whom had detectable ctDNA in their TISF prior to bevacizumab administration. The top columns represent the number of mutations carried by patients; the side columns represent the number of patients carrying a particular mutation; the bottom columns represent patient characteristics, including age, sex, and tumour location. (B) Gene‐related signalling pathways cleared in TISF samples after adding bevacizumab in seven patients. (C) Acquisition of gene‐associated signalling pathways in TISF samples after adding bevacizumab in seven patients. (D) Change in ctDNA levels in TISF samples at relapse and 3 months after adding bevacizumab in 10 patients. All 10 patients showed a reduction or disappearance of areas of enhancement on imaging MRI and a significant improvement in clinical symptoms following the use of bevacizumab, but there was no significant difference in ctDNA levels before (median maximum variant allele frequencies [MVAF], 4.55%) and after (median MVAF, 3.34%; *p* = .1309) treatment with bevacizumab, which may be related to the fact that although bevacizumab was effective in improving patients’ clinical symptoms and prolonging PFS, it was not effective in stopping the progression of tumour at disease progression.^7^, ^8^ The middle black line is at the mean; the lower and upper half‐lines indicate error lines. ns, not significant; MUT, mutant

In this study, longitudinal analysis of gliomas genomic changes provides more conclusive evidence supporting ctDNA sequencing in TISF to capture the mutational gene profile in gliomas dynamically. What is more, we have revealed through serial ctDNA analysis that gliomas have a high degree of temporal heterogeneity in their true in vivo evolution under standard therapeutic pressure and have identified significant differences in survival prognosis for patients with altered mutational status in genes like GNAS and CIC, which can be used as biological markers to provide early and accurate molecular risk stratification. Changes in ctDNA‐related level indicators such as MVAF and the number of mutations can indicate intra‐tumour heterogeneity^9^ and assess the true picture of PD at the molecular level. In conclusion, dynamic monitoring of the genome of glioma patients undergoing standard treatment can facilitate the precise management of glioma patients and clinical management and research.

## CONFLICT OF INTEREST

The authors declare no potential conflicts of interest.

## Supporting information

Supporting Information

Supporting Information

Supporting Information

Supporting Information

Figure S1 Flow chart of study design and patient screening
Figure S2 Genetic characterization of primary tumor tissue in 33 patients and time from postoperative to imaging suggestive of disease progression (PD) in 20 patients.
Figure S3 (A and B) The heat map compares the VAF of high‐frequency‐mutated genes in TISF samples at disease progression in Patient 1 and Patient 15, respectively, with the VAF of the corresponding loci in recurrent tumour tissue samples from recurrent secondary surgery.
Figure S4 ctDNA changes in TISF and tumour tissue suggest two patterns of tumour recurrence evolution.

Table S1 Clinical characteristics of 33 patients with glioma at disease progression
