# Supplementary figures and images for: Real‐time longitudinal analysis of human gliomas reveals in vivo genome evolution and therapeutic impact under standardized treatment
Source: Clin Transl Med. 2022 Jul 8;12(7):e956. doi: 10.1002/ctm2.956 (PMC9269997; doi:10.1002/ctm2.956)

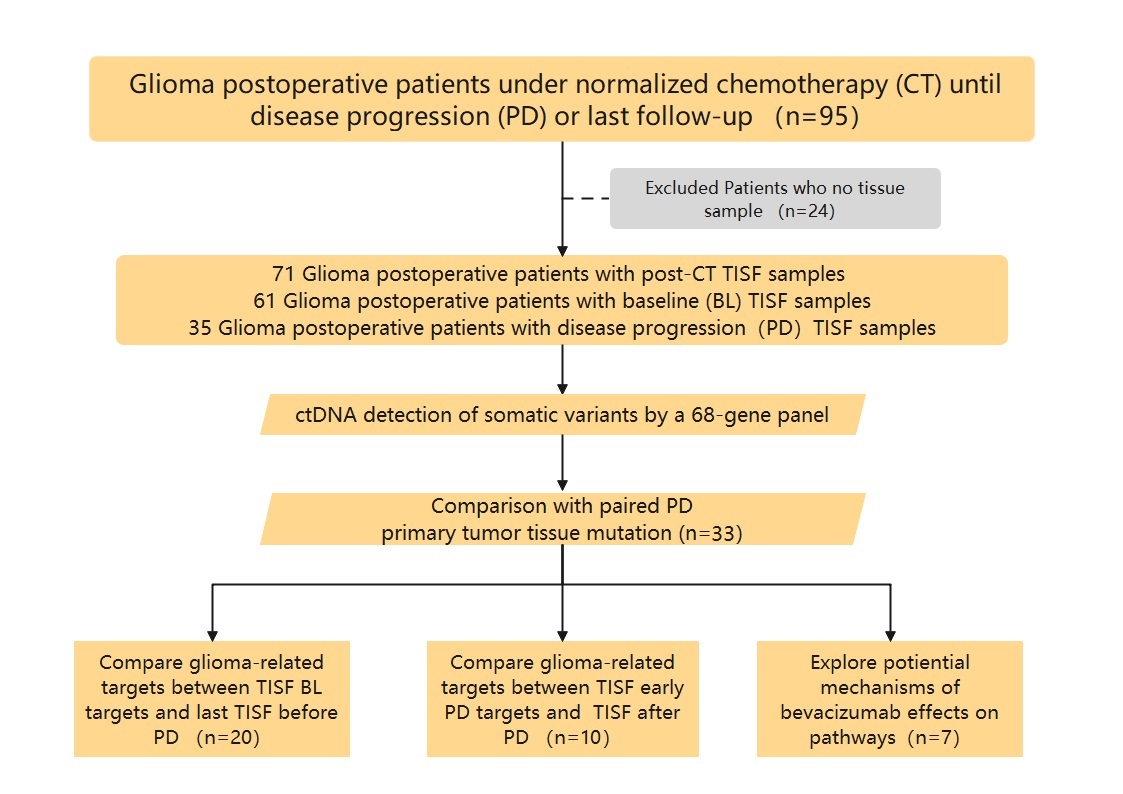

Supplement: Supplementary file 1 — Supporting Information [file CTM2-12-e956-s004.jpg]

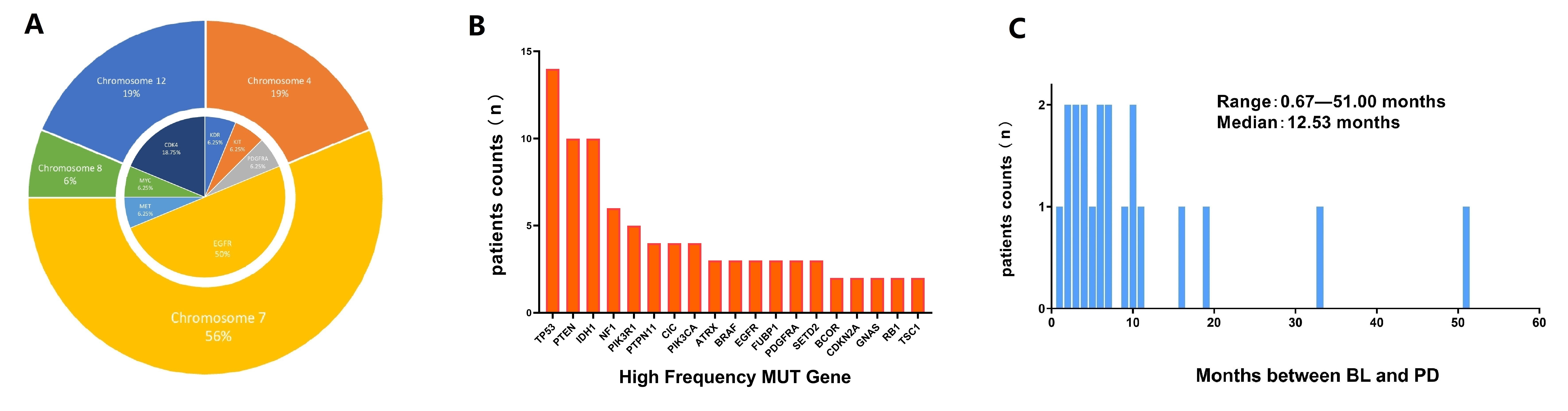

Supplement: Supplementary file 2 — Supporting Information [file CTM2-12-e956-s005.jpg]

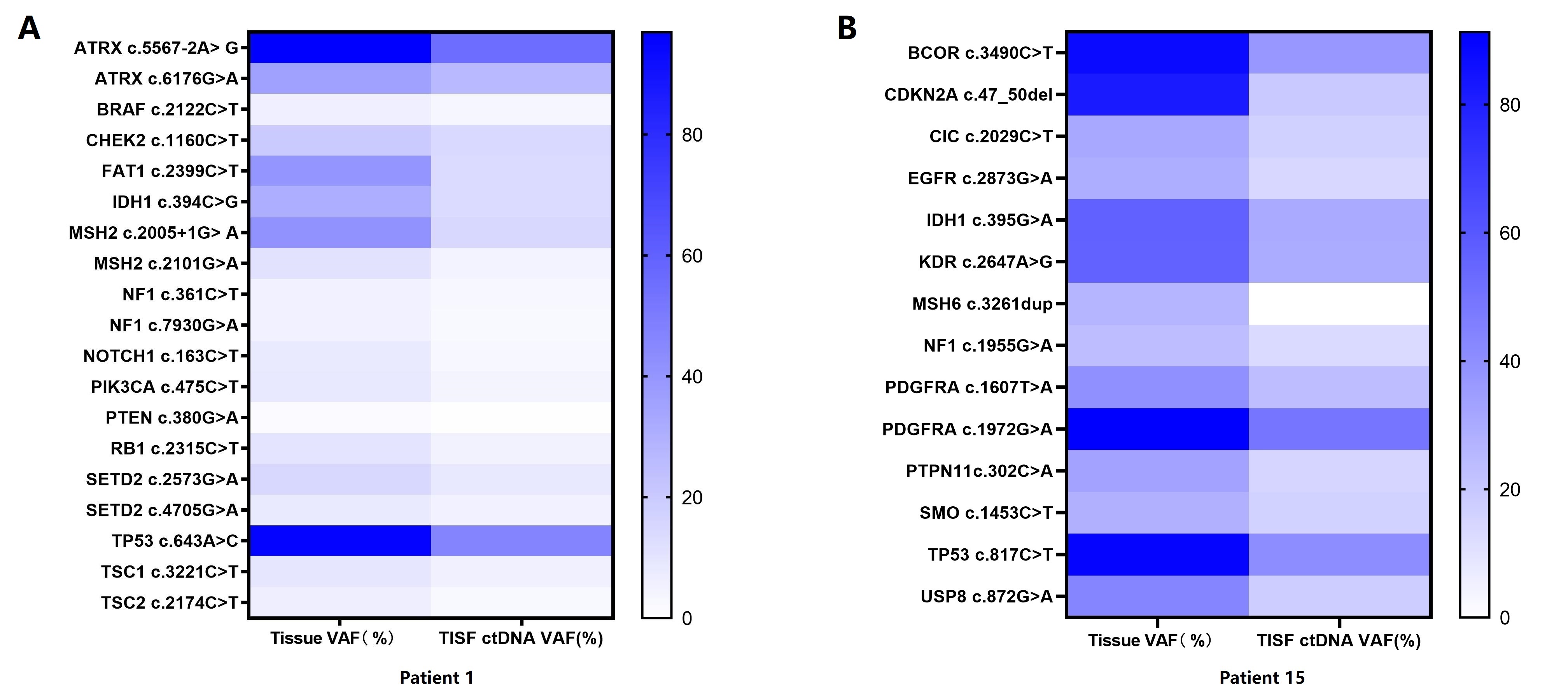

Supplement: Supplementary file 3 — Supporting Information [file CTM2-12-e956-s001.jpg]

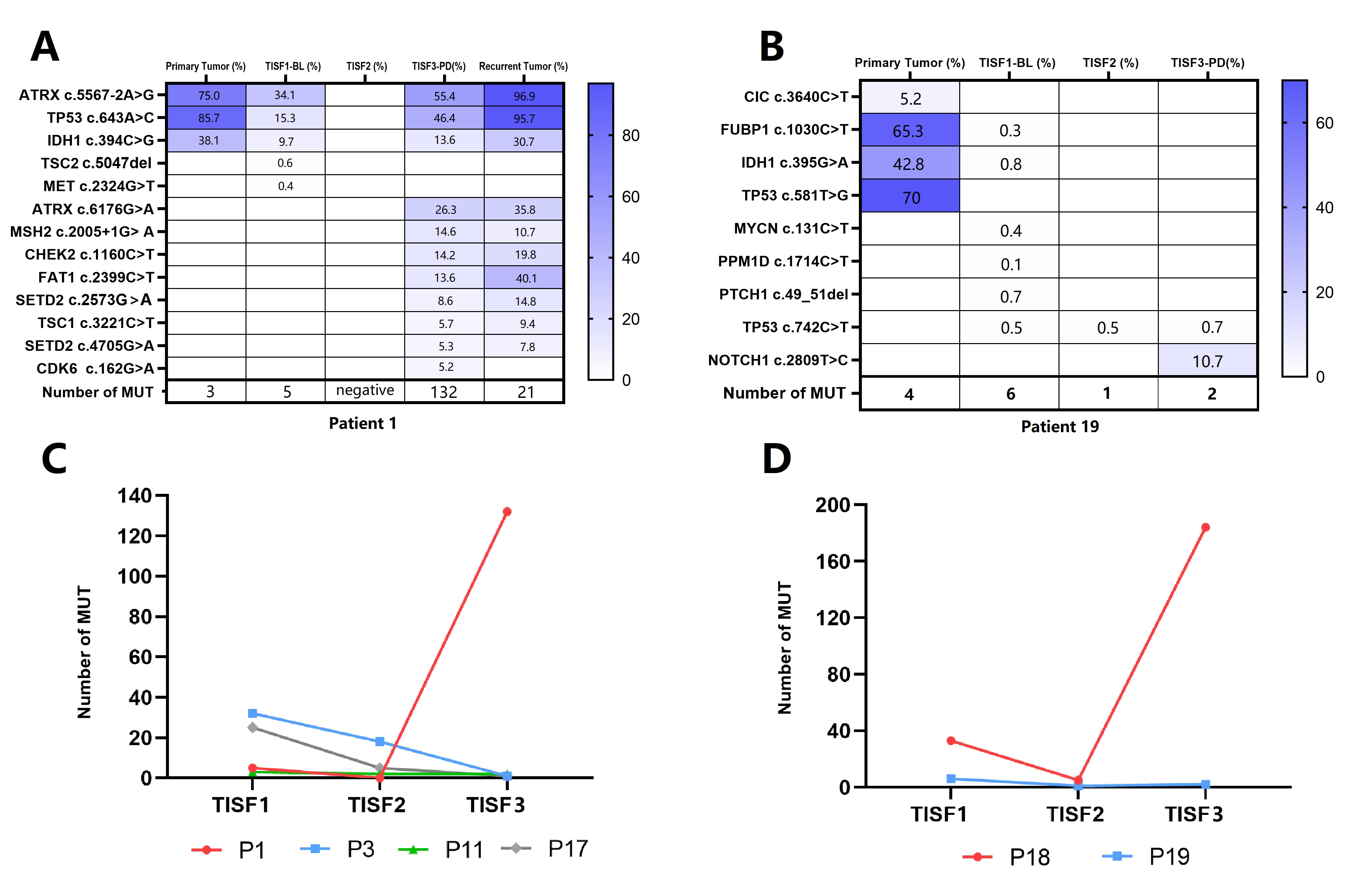

Supplement: Supplementary file 4 — Supporting Information [file CTM2-12-e956-s006.jpg]
