# Supplementary material for: Real‐time longitudinal analysis of human gliomas reveals in vivo genome evolution and therapeutic impact under standardized treatment
Source: Clin Transl Med. 2022 Jul 8;12(7):e956. doi: 10.1002/ctm2.956 (PMC9269997; doi:10.1002/ctm2.956)
Supplement: Supplementary file 5 — Figure S1 Flow chart of study design and patient screening Figure S2 Genetic characterization of primary tumor tissue in 33 patients and time from postoperative to imaging suggestive of disease progression (PD) in 20 patients. Figure S3 (A and B) The heat map compares the VAF of high‐frequency‐mutated genes in TISF samples at disease progression in Patient 1 and Patient 15, respectively, with the VAF of the corresponding loci in recurrent tumour tissue samples from recurrent secondary surgery. Figure S4 ctDNA changes in TISF and tumour tissue suggest two patterns of tumour recurrence evolution. [file CTM2-12-e956-s003.docx]

**Supporting Information**

**Figure S1. Flow chart of study design and patient screening.**

ctDNA, circulating tumor DNA; TISF, Tumor In Situ Fluid；CT，chemotherapy；BL，baseline；PD，progressive disease.

**Figure S2.** **(A)** Gene amplification in primary tumor tissue. In the primary tumor tissue of 33 patients, gene amplification occurred in 33.3% (11/33) patients. Of these amplified genes, 56% occurred on chromosome 7 for EGFR and MET, respectively, and the rest occurred on chromosomes 4 (19%), 12 (19%), and 8 (6%), respectively. **(B)** High-frequency mutated genes are detected in the primary tumor tissue. **(C)** Time from patient's postoperative period to imaging suggestive of disease progression (PD). In the cohort of 20 patients with serial TISF samples collected prior to disease progression, the median time from baseline ctDNA collection after surgery to the time of disease progression was 6.27 months (range, 0.67-51.00 months).

**Figure S3.** **(A) (B)** The heat map compares the VAF of high-frequency mutated genes in TISF samples at disease progression in Patient 1 and Patient 15, respectively, with the VAF of the corresponding loci in recurrent tumor tissue samples from recurrent secondary surgery. We found that mutated genes with larger VAF in recurrent tumor tissue also had larger VAF in TISF at disease progression with the same mutation loci.

**Figure S4.** **ctDNA changes in TISF and tumor tissue suggest two patterns of tumor recurrence evolution. (A)** The heat map represents the mutations and VAF (%) found in the tumor DNA and TISF ctDNA samples from Patient 1. **(B)** The heat map represents the mutations and VAF (%) found in the tumor DNA and TISF ctDNA samples from Patient 19. **(C)(D)** Changes in the number of mutated genes in TISF from BL to PD in 4 and 2 patients. Patients11,1,3,17 showed a trend of decreasing and then increasing MVAF and tumor volume load during disease progression (Figure3 C), and the corresponding number of mutations showed a decreasing trend (except for Patient 1, where hypermutation occurred and the number of mutations increased significantly), suggesting that in the early stage of treatment, the treatment effect was better, the volume load decreased, the non-resistant clones decreased, and MVAF, tumor volume load, and the mutation number decreases. With the accumulation of time, the same dominant mutations in the tumor as in the original tumor occupied the main body, and the tumor cells proliferated, causing the volume load to rising and the volume to increase, leading to the recurrence of the tumor; while MVAF and tumor volume of Patient 18,19 showed an increasing trend, and the change in the number of mutations showed a decreasing and then increasing trend, suggesting that in the early stage of treatment, the tumor had produced various subclones (part of them were drug resistance-associated subclones), and under the pressure of treatment, tumor cells containing drug resistance-associated subclones were preserved (10), while others were eliminated, resulting in a decrease in the number of mutations. However, these tumor cells containing drug-resistant-associated subclones continue to proliferate and gradually dominate from the early stage of treatment, keeping the tumor volume load, and MVAF elevated and leading to tumor recurrence.
