# Supplementary material for: Real‐time longitudinal analysis of human gliomas reveals in vivo genome evolution and therapeutic impact under standardized treatment
Source: Clin Transl Med. 2022 Jul 8;12(7):e956. doi: 10.1002/ctm2.956 (PMC9269997; doi:10.1002/ctm2.956)
Supplement: Supplementary file 6 — Table S1 Clinical characteristics of 33 patients with glioma at disease progression [file CTM2-12-e956-s002.docx]

**Supporting Information**

**Supplemental Table S1. Clinical characteristics of 33 patients with glioma at disease progression**

| **Characteristics** |  |  |  | No.（%） |
| --- | --- | --- | --- | --- |
| **All Patients, No.** | |  |  | 33 |
| **Age (median, range)** | |  |  | 57（26，78） |
| **Gender** | |  |  |  |
| Female | |  |  | 14（42.42） |
| Male | |  |  | 19（57.58） |
| **Left/Right** | |  |  |  |
| Left-sided | |  |  | 17（51.52） |
| Right-sided | |  |  | 16（48.48） |
| **Primary Tumor Location** | |  |  |  |
| frontal lobe | |  |  | 14（42.42） |
| occipital lobe | |  |  | 2 （6.06） |
| parietal lobe |  |  |  | 12（36.36） |
| temporal lobe |  |  |  | 19（57.58） |
| insular lobe |  |  |  | 3 （9.09） |
| Others* |  |  |  | 9 （27.27） |
| **Pathological Type** | |  |  |  |
| glioblastoma | |  |  | 22（66.67） |
| astrocytoma | |  |  | 6 （18.18） |
| oligodendroglioma | |  |  | 5 （15.15） |
| **GNAS/CIC Status in TISF** | |  |  |  |
| GNAS mutant | |  |  | 2 （6.06） |
| CIC mutant  GNAS &CIC mutant | |  |  | 4 （12.12）  5 （15.15） |
| GNAS &CIC wild | |  |  | 22（66.67） |

*Others Primary Tumor Location include corpus callosum and cerebellum.
